# The Effect of Temperature on *Drosophila* Hybrid Fitness

**DOI:** 10.1534/g3.116.034926

**Published:** 2016-12-02

**Authors:** Charles J. J. Miller, Daniel R. Matute

**Affiliations:** Biology Department, University of North Carolina, Chapel Hill, North Carolina 27510

**Keywords:** postzygotic isolation, hybrids, *Drosophila*, temperature

## Abstract

Mechanisms of reproductive isolation inhibit gene flow between species and can be broadly sorted into two categories: prezygotic and postzygotic. While comparative studies suggest that prezygotic barriers tend to evolve first, postzygotic barriers are crucial for maintaining species boundaries and impeding gene flow that might otherwise cause incipient species to merge. Most, but not all, postzygotic barriers result from genetic incompatibilities between two or more loci from different species, and occur due to divergent evolution in allopatry. Hybrid defects result from improper allelic interactions between these loci. While some postzygotic barriers are environmentally-independent, the magnitude of others has been shown to vary in penetrance depending on environmental factors. We crossed *Drosophila melanogaster* mutants to two other species, *D. simulans* and *D. santomea*, and collected fitness data of the hybrids at two different temperatures. Our goal was to examine the effect of temperature on recessive incompatibility alleles in their genomes. We found that temperature has a stronger effect on the penetrance of recessive incompatibility alleles in the *D. simulans* genome than on those in the *D. santomea* genome. These results suggest that the penetrance of hybrid incompatibilities can be strongly affected by environmental context, and that the magnitude of such gene-by-environment interactions can be contingent on the genotype of the hybrid.

Reproductive barriers hamper gene flow between species (Coyne and Orr 2004). Depending on when in the reproductive cycle barriers occur, they can be classified as prezygotic or postzygotic. Phenotypes that prevent the successful formation of a zygote, such as certain behavioral or gametic incompatibilities, can lead to prezygotic isolation (reviewed in Coyne and Orr (2004)). Conversely, postzygotic isolation manifests as defects in hybrids and includes a range of phenotypic defects such as developmental breakdown and behavioral abnormalities (reviewed in Maheshwari and Barbash (2011)). In its most extreme form, postzygotic isolation results in hybrid inviability (HI).

The evolution of postzygotic isolation is crucial to speciation for at least three reasons. First, even though comparative studies have strongly suggested that prezygotic isolation tends to evolve faster than postzygotic isolation (Coyne and Orr 1989; Orr *et al.* 1997; Mendelson 2003; Rabosky and Matute 2013), they are often not strong enough to prevent the fusion of nascent species (Rosenblum *et al.* 2012; Comeault *et al.* 2015; Cenzer 2016). Postzygotic barriers are more robust and are often crucial to maintaining separation of species. Second, hybrid defects can also influence the evolution of other barriers to gene flow (reviewed in Servedio and Noor 2003; Hopkins 2013). For example, in the process of reinforcement, prezygotic isolation becomes stronger in areas of sympatry due to indirect selection on hybrids with deleterious phenotypes (Servedio and Noor 2003; Hudson and Price 2014). Finally, studying postzygotic isolation, and other traits that reduce fitness in hybrids, can reveal how much functional divergence has occurred between the genomes of the parent species, furthering our understanding of the processes that initiate and maintain separation of species (Coyne and Orr 2004; Orr *et al.* 2007; Rosenblum *et al.* 2012).

Postzygotic isolation frequently results from Dobzhansky–Muller incompatibilities (DMIs, reviewed in Coyne and Orr 2004; and Nosil and Schluter 2011). According to the Dobzhansky–Muller model, deleterious epistatic interactions between alleles from different species reduce fitness in hybrids (Muller *et al.* 1937; Dobzhansky *et al.* 1942; Orr 1995; Coyne and Orr 2004). The model requires at least two interacting loci that evolve separately in allopatric populations. Postzygotic isolation arises as a collateral effect when the species come into secondary contact and hybridize. For example, the ancestral alleles at a pair of loci are “a-b,” and two allopatric populations subsequently diverge into “a-B” and “A-b.” The hybrid genotype “A-B” has deleterious consequences due to the interaction between the “A” and “B” alleles, which were only present together in the hybrid. DMIs are frequently implicated in the defects observed in many interspecies hybrids, and thus are crucial to understanding how species form and persist over time.

Several mapping efforts have succeeded in characterizing the developmental defects underlying HI, as well as the causal alleles in some cases (reviewed in Nosil and Schluter 2011; Maheshwari and Barbash 2012; Sawamura 2016). These studies have revealed two general trends regarding the evolution of postzygotic isolation. First, sex chromosomes frequently harbor alleles that lead to sterility and inviability in hybrids (Masly and Presgraves 2007; Carrington *et al.* 2011), which may explain a pattern known as “Haldane’s rule”: when hybrids have a defect, the heterogametic sex is typically more severely affected (Orr *et al.* 1997; Delph and Demuth 2016). Second, hybrid incompatibilities accumulate at an exponential rate through a process known as the snowball theory, a key prediction of the Dobzhansky–Muller model (Orr 1995; Orr and Turelli 2001; Matute *et al.* 2010; Moyle and Nakazato 2010; Matute and Gavin-Smyth 2014; Wang *et al.* 2015).

*Drosophila* hybrids have been crucial for our understanding of the genetic basis of HI (Orr *et al.* 2007; Aruna *et al.* 2009). In particular, the study of crosses between *Drosophila melanogaster* females and *D. simulans* males has been one of the most informative for investigating the genetic basis of postzygotic isolation. *D. simulans* is thought to have originated in Southeast Africa, is widespread around the globe, and has a similar thermal tolerance and niche preference to *D. melanogaster* (Stanley *et al.* 1980; Austin and Moehring 2013). Interspecific crosses between *D. melanogaster* females and *D. simulans* males produce only sterile hybrid females; male offspring die as larvae (Sturtevant 1920; Inoue and Watanabe 1979). The genetic basis of hybrid male lethality has been finely mapped and at least three loci, one on each major chromosome, have been found to be involved in the epistatic interaction responsible for male HI. Different alleles are fixed in the gene triad *Hmr/Lhr/gfzf* between *D. melanogaster* and *D. simulans*, and their interaction in hybrid offspring is deleterious (Barbash *et al.* 2000; Phadnis *et al.* 2015; Cooper and Phadnis 2016). Additionally, two alleles influencing the viability of hybrid females have also been mapped: *Nup96* (Presgraves 2003) and *Nup160* (Tang and Presgraves 2009).

*D. melanogaster* can also hybridize with species to which it is even more distantly related than *D. simulans* (Matute *et al.* 2009a; Matute *et al.* 2010). The cross between *D. melanogaster* and *D. santomea* also produces only hybrid females (Matute *et al.* 2009a); males fail to develop the distal half of the abdomen and die as embryos (Gavin-Smyth and Matute 2013, Matute and Gavin-Smyth 2014). This cross is the most divergent known to produce hybrid progeny in *Drosophila* (Matute *et al.* 2010). *D. santomea* is endemic to the highlands of São Tomé, a volcanic island off the coast of Cameroon (Lachaise *et al.* 2000). On the extinct volcano of Pico de São Tomé, *D. santomea* occupies the mist forests of the island at high elevations, where it is thought to breed on figs of the endemic subspecies *Ficus chlamydocarpa fernandesiana* (Lachaise *et al.* 2000; Llopart *et al.* 2005a,b). Within the *D*. *melanogaster* species subgroup, *D. santomea* and *D. simulans* have very different life history traits, whereas *D. simulans* and *D. melanogaster* are more similar (Capy and Gibert 2004). For example, *D. melanogaster* and *D. simulans* are both globally distributed (Capy and Gibert 2004), but *D. santomea* is restricted to the high altitudes of São Tomé. Similarly, *D. melanogaster* and *D. simulans* are temperature generalists, while *D. santomea* is a temperature specialist.

In previous studies of hybrids between *D. melanogaster* and *D. simulans*, the penetrance of a few HI alleles has been found to be largely, but not completely, independent of environmental factors (Barbash *et al.* 2000; Presgraves *et al.* 2003; Tang and Presgraves 2009). Nonetheless, other HI loci might be affected by extrinsic factors (Coyne *et al.* 1998; Presgraves *et al.* 2003). For example, temperature has been shown to affect the magnitude of HI in several clades (*Tribolium* beetles: Wade *et al.* 1999; Dowling *et al.* 2007; *Nasonia* wasps: Bordenstein *et al.* 2001; Koevoets *et al.* 2012; *Nicotiana*: Yamada *et al.* 2000; Muralidharan *et al.* 2014). Crosses between *D. melanogaster* and *D. simulans* have been used to identify genomic regions in *D. simulans* associated with HI at two different temperatures (Coyne *et al.* 1998). Similarly, hybrids between *D. melanogaster* and *D. mauritiana* have revealed that alleles from *D. melanogaster* may also have different effects at different temperatures (Cattani and Presgraves 2012). Finally, temperature-dependent rescue of male inviability by mutant *Hmr* has been shown in hybrids of *D. melanogaster* with both *D. simulans* and *D. mauritiana* (Hutter and Ashburner 1987). However, we know little regarding whether the same type of variance in penetrance occurs in other interspecific hybrids.

We tested whether environmentally-dependent inviability can be observed in two *Drosophila* interspecific hybrids: *D. melanogaster/D. santomea* F1 females (*mel/san*) and *D. melanogaster/D. simulans* F1 females (*mel/sim*). Given that *D. santomea* is a temperature specialist (Matute *et al.* 2009a) and *D. simulans* is a generalist (Capy and Gibert 2004), we explored whether the penetrance of recessive inviability alleles in hybrids with *D. melanogaster* was affected by temperature. Our expectation was that HI should be strongly affected by both the identity of the species involved in the interspecific crosses and the temperature at which hybrids developed. We hypothesized that *mel/san* hybrids would be much more strongly affected by temperature than *mel/sim* hybrids. Our results indicate that, even though the penetrance of particular loci is affected by temperature in both the *mel/san* and *mel/sim* crosses, HI is more affected by temperature in *mel/sim* hybrids than in *mel/san* hybrids.

## Materials and Methods

We crossed *D. melanogaster* females carrying a chromosomal deficiency with either *D. simulans* or *D. santomea* males in order to map recessive hybrid incompatibility alleles. Larvae were reared at 18°. We compared the results of our mapping with a previous study that identified HI loci at 24° for these two species pairs. We describe each step as follows.

### Species and stocks

We used one outbred stock for *D. santomea* and one for *D. simulans*. These stocks were generated by combining males and females from multiple isofemale lines. The *D. santomea* stock SYN2005 was generated by mixing six isofemale lines collected in the highlands of São Tomé. *D. simulans* FC was created by J. Coyne and has been previously reported (Coyne *et al.* 1998; Matute and Gavin-Smyth 2014). All lines were reared on standard cornmeal/Karo/agar medium at 24° under a 12 hr light/dark cycle in 100 ml bottles. Adults were allowed to oviposit for 1 wk, after which time the bottles were cleared. We added 1 ml of propionic acid (0.5% v/v) solution to the vials and provided a pupation substrate (Kimwipes Delicate Task; Kimberly Clark, Irving, TX). At least 10 bottles of each species were kept in parallel to guarantee the collection of large numbers of virgins.

*D. melanogaster* deficiency stocks were purchased from the Bloomington *Drosophila* Stock Center in five batches, one for each chromosomal arm. Once quarantined, stocks were expanded in 200 ml plastic bottles containing cornmeal food. We let females oviposit; when larvae were observed in the bottles, they were monitored daily for black pupae. All flies were kept at 24° under a 12 hr light/dark cycle. Supplemental Material, Table S1 lists all the stocks used in this report.

### Virgin collection

To cross *D. melanogaster* deficiency stocks to male *D. santomea* or *D. simulans*, we needed virgin females from each *D. melanogaster* mutant stock. We kept *D. melanogaster* deficiency stocks in 300 ml plastic bottles with cornmeal fly food. Once dark pupae were observed, bottles were cleared every 12 hr. Females from these mutant stocks were collected as virgins within 8 hr of eclosion under CO_2_ anesthesia and kept for 3 d in single-sex groups of 20 flies in 30 ml corn meal food-containing vials. Males were also collected daily from kimwiped bottles but were not necessarily virgins. They were kept in all-male vials (20 individuals per vial). On day four, we assessed whether there were larvae in the media in both the female and male vials. If the inspection revealed any progeny, the vial was discarded. If the vials had no larvae, the virgin individuals were used for crosses.

### Deficiency mapping

**Figure 1:**
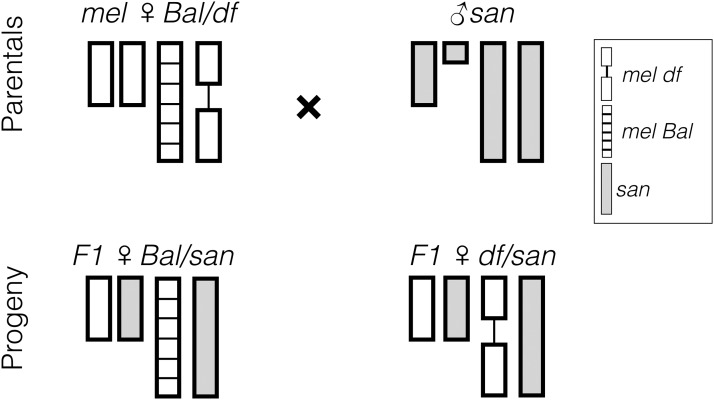
Deficiency mapping approach to detect alleles involved in hybrid inviability. A significant dearth of *df/san* individuals compared to their *Bal/san* sisters indicates that the deficiency uncovers a lethal or semilethal allele involved in hybrid inviability. *D. melanogaster* balancer chromosomes are shown as striped bars; deficiency chromosomes are shown as a line connecting two bars. *D. santomea* chromosomes are shown in light gray. Sex chromosomes are shown as shorter bars than autosomes, and *Y* is shown as shorter than the *X*. *Bal*, balancer chromosome; *df*, deficiencies; *mel*, *D. melanogaster*; *san*, *D. santomea*.

We used deficiency mapping to detect recessive alleles from the *D. santomea* genome involved in HI (Coyne *et al.* 1998; Presgraves 2003). Our crossing design detects recessive partners of a DMI in a species crossable with *D. melanogaster* by uncovering recessive deleterious alleles with null alleles of a genomic region from *D. melanogaster*. The approach involves crossing females from *D. melanogaster* (*mel*) stocks containing known genomic deletions, or “deficiencies” (*df*, Bloomington *Drosophila* Stock Center), maintained as heterozygotes against a balancer (*Bal*) chromosome carrying a dominant homozygous lethal mutation, to *D. santomea* (*san*) males (Figure 1). On day four after virgin collection, males and females were mixed in a 30 ml plastic vial with cornmeal fly food. The ratio of females to males was always 1:2 and at least 10 females were used per cross. To maximize the lifespan of flies, we maintained all crosses with the vial lying on its side for the duration of the assay. Vials were inspected every 5 d to check for progeny. We transferred the parents to a new vial when we observed either larvae or dead embryos. The old vial was tended by dampening the media with propionic acid and adding tissue paper (Kimwipes, Kimtech Science) for the larvae to pupate upon. We performed at least 20 replicates per cross and on average 10 of them produced progeny. Crosses were kept until no more progeny were produced from each vial.

### Assessment of HI

**Figure 2:**
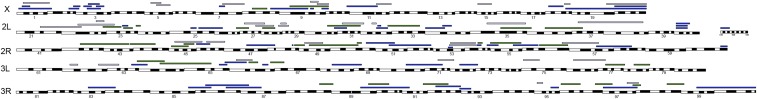
Deficiency mapping of hybrid incompatibilities in the *D. santomea* genome at two different temperatures. Light blue: hybrid inviability only at 18°. Green: hybrid inviability only at 24°. Dark blue: hybrid inviability at both temperatures. Deficiencies not causing hybrid inviability are not shown.

We measured the effect of each hemizygous region (those expressing *san or sim* recessive alleles) on the viability of hybrid female offspring (Figure 2). If a *D. melanogaster* deficiency uncovered a completely lethal recessive region of the *D. santome*a genome (one which caused lethality in F_1_ hybrids), this cross would produce *Bal/san* but not *df/san* hybrid females (Figure 1, Coyne *et al.* 1998; Matute *et al.* 2010). If the *D. melanogaster* deficiency uncovered a recessive region of the *D. santomea* genome that compromised hybrid fitness but did not cause complete lethality, then this cross would produce an excess of *Bal/san* compared to *df/san* hybrid females (as assessed by a χ^2^ test, 1 degree of freedom). Cases in which *Bal/san* hybrids are significantly more common than *df/san* hybrids indicate epistatic interactions between a recessive *san* allele (exposed when hemizygous) and a dominant factor in the *mel* genome (Coyne *et al.* 1998). This allowed us to measure HI quantitatively instead of as a binary trait. All crosses were kept at 18° once started.

### Counting HI alleles

The minimal number of HI alleles was determined by counting the number of overlapping deficiencies associated with HI. If two deficiencies overlap and both cause HI, it can be assumed that they share a locus involved in HI. To assess whether the density of hybrid incompatibility alleles was uniform across chromosomal arms, we compared the observed number of hybrid incompatibility alleles with the expectations from a uniform distribution (*i.e.*, same number of hybrid incompatibilities alleles in the five chromosomal arms) using Pearson’s χ^2^ test with simulated *P* values (based on 2000 replicates, library “stats”; R Core Team 2014).

### Effect of temperature

To assess whether temperature affected the viability of different hybrid genotypes, we measured HI at 18° and compared it with the magnitude of HI at 24° [data for HI at 24° were previously published in Matute *et al.* (2010)]. In order to minimize the effect of different genetic backgrounds, we only compared HI between stocks that had been evaluated at both temperatures and in both hybrid crosses. First, we compared the mean viability of *df*-carrying hybrid individuals of each genotype (*i.e.*, deficiency) at the two temperatures using paired *t*-tests. We did two tests, one for each interspecific cross (R, library “stats”; R Core Team 2014).

Next, we fitted two linear models to the data in order to analyze the interaction between hybrid genotype, deficiency, and temperature. First, we fitted a linear model in which the viability of the *df*-carrying genotype was the response; the temperature (18 and 24°) and the hybrid genotype (*mel/sim* and *mel/san*) were fixed effects. We also included the interaction between temperature and hybrid genotype. The linear model followed the form:

$$\mathrm{viab}{\left(\mathrm{df}\right)}_{i}\;∼\mathrm{tem}p_{i}\;+\mathrm{genotyp}e_{j}\;+\mathrm{temp}\times \mathrm{genotyp}e_{\mathrm{ij}}\;+\mathrm{Erro}r_{\mathrm{ij}}$$

The linear model was fitted with the function “lm” (R, library “stats”; R Core Team 2014). Pairwise *post hoc* comparisons were done with a Tukey Honest Significant Difference (HSD) test using the function “glht” (R, library “multcomp”; Hothorn *et al.* 2008). We also fitted a linear model that included only the interaction between temperature and genotype:

$$\mathrm{viab}{\left(\mathrm{df}\right)}_{i}\;∼\mathrm{temp}\times \mathrm{genotyp}e_{\mathrm{ij}}\;+\mathrm{Erro}r_{\mathrm{ij}}$$

### Effect of chromosome

We fitted a linear model to test whether the sex chromosomes and autosomes had different effects on HI. Since we were only interested in assessing whether temperature affected the fitness of *df*-carrying hybrids differently in the two hybrids, the linear model had three fixed effects: location (chromosome), temperature (18 and 24°), and genotype (*mel/san* and *mel/sim*). The model also included all possible interactions between the effects:

$$\mathrm{viab}{\left(\mathrm{df}\right)}_{i}∼\mathrm{chromosome}{\;}_{i}+\mathrm{temperatur}e_{j}+\mathrm{genotyp}e_{k}+\mathrm{chromosome}\times \mathrm{temperatur}e_{\mathrm{ij}}+\mathrm{chromosome}\times \mathrm{genotyp}e_{\mathrm{ik}}+\mathrm{temperature}\times \mathrm{genotyp}e_{\mathrm{jk}}+\mathrm{chromosome}\times \mathrm{temperature}\times \mathrm{genotyp}e_{\mathrm{ijk}}\;+\mathrm{Erro}r_{\mathrm{ijk}}$$

The model was fitted with the function “lm” (R, library “stats”; R Core Team 2014). Pairwise comparisons were done with a Tukey HSD test using the function “glht” (R, library multcomp; Hothorn *et al.* 2008) in a linear model that only included the interaction effect between deficiency location, temperature, and genotype:

$$\mathrm{viab}{\left(\mathrm{df}\right)}_{i}∼\mathrm{chromosome}\times \mathrm{temperature}\times \mathrm{genotyp}e_{\mathrm{ijk}}\;+\mathrm{Erro}r_{\mathrm{ij}}k$$

### Data availability

Table S1 contains all the raw data for this contribution. Data and analytical code were also deposited in Dryad (doi: 10.5061/dryad.511ms).

## Results

We identified the genomic regions containing recessive hybrid incompatibilities in the genomes of hybrids between *D. melanogaster* and either *D. santomea* or *D. simulans* when those hybrids were reared at 18°. Recessive hybrid incompatibility alleles at 24° were previously mapped in both hybrids (*mel/san* and *mel/sim*; Matute *et al.* 2010). We first report the results for each species independently and then compare the results between species and temperatures.

### D. santomea

We used 223 *D. melanogaster* deficiency stocks (spanning 78.22% of euchromatic regions) and found 91 that caused partial or complete hybrid incompatibility when crossed to *D. santomea* at 18° (Figure 2). We compared our results with the map of inviability alleles at 24°, where 90 deficiencies caused HI. We found that the slight plurality of deficiencies (56 deficiencies) caused HI at both 24 and 18°. Thirty-five regions cause inviability only at 18° and 34 regions cause inviability only at 24°. The overlap of incompatibilities between temperatures was significant (randomization tests: *P* < 1 × 10^−4^; Table S2). The same result is found if we assess the effect of temperature for the minimum number of hybrid incompatibilities (correcting for overlapping deficiencies, which may share a common deficiency rather than represent several unique deficiencies). We found that the slight plurality of regions (43 regions) caused HI at both 24 and 18°. Twenty-nine regions cause inviability only at 18° and 31 regions cause inviability only at 24°. The overlap of incompatibilities between temperatures was also significant (randomization tests: *P* < 1 × 10^−4^). This is particularly interesting because we find the opposite pattern in the *mel/sim* cross (see below).

We found no difference in the relative density of incompatibilities across chromosomes. This was true for loci that cause HI at only 18° (χ^2^ = 2.6154, *P* = 0.913), at only 24° (χ^2^ = 3.587, *P* = 0.609), and at both temperatures (χ^2^ = 0.789, *P* = 0.977).

### D. simulans

We used the same panel of 223 *D. melanogaster* deficiencies to detect hybrid incompatibilities in the *D. simulans* genome. At 18°, we found seven deficiency stocks that caused partial or complete hybrid incompatibility when crossed to *D. simulans* (Figure 2). We compared these results with the map of inviability alleles at 24°, where 17 deficiencies lead to HI. Of the previously reported deficiencies that uncovered hybrid incompatibilities, we found that 16 of these 17 regions caused HI at only 24° and not at 18°. There was no significant overlap of incompatibilities between temperatures (randomization tests: *P* < 0.4312; Table S3). The same result is found when we assess the effect of temperature on the minimal number of hybrid incompatibility regions (correcting for overlapping regions of deficiencies which may all uncover the same recessive lethal allele): only one region causes HI at both temperatures; fourteen hybrid incompatibility alleles cause HI at only 24°. Notably, we found a group of six deficiencies that only cause HI at 18°, which corresponds to at least five hybrid incompatibility regions.

#### Effect of temperature in HI alleles:

**Figure 3:**
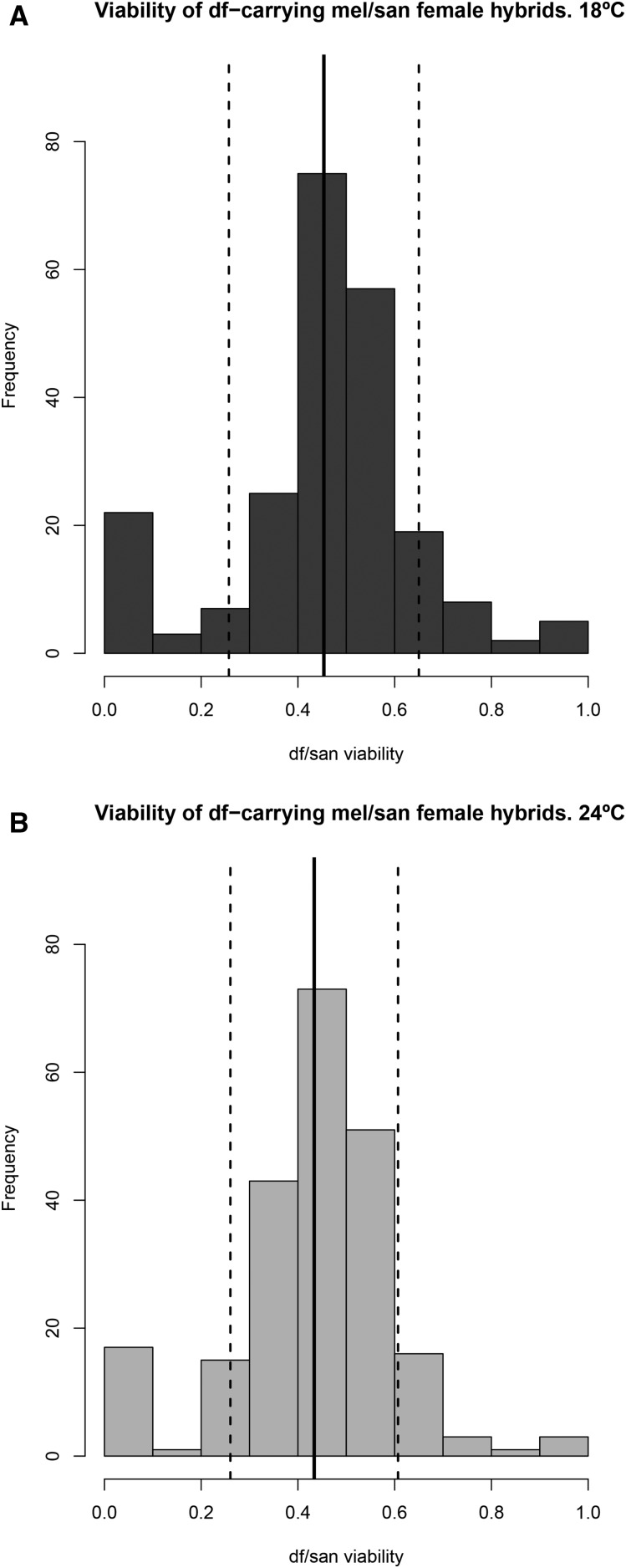
Relative fitness frequencies of the *df*-carrying hybrids in *mel/san* hybrids at two different temperatures. (A) *mel/san* 18°. (B) *mel/san* 24°. Black solid lines in each panel show the mean fitness of the *df*-carrying hybrids. Black dashed lines show the mean ± SD of the mean. The *x*-axis shows relative viability of deficiency-carrying progeny (observed *df*-carrying progeny/observed *Bal*-carrying progeny + observed *df*-carrying progeny) while the *y*-axis shows the number of stocks having a given level of viability of deficiency carrying offspring. *Bal*, balancer chromosome; *df*, deficiencies; *mel*, *D. melanogaster*; *san*, *D. santomea*.

**Figure 4:**
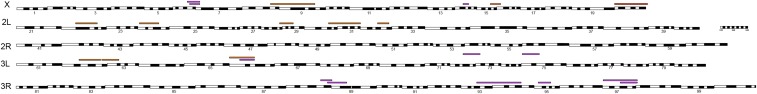
Deficiency mapping of hybrid incompatibilities in the *D. simulans* genome at two different temperatures. Orange: hybrid inviability only at 18°. Pink: hybrid inviability only at 24°. Red: hybrid inviability at both temperatures. Deficiencies not causing hybrid inviability are not shown.

**Figure 5:**
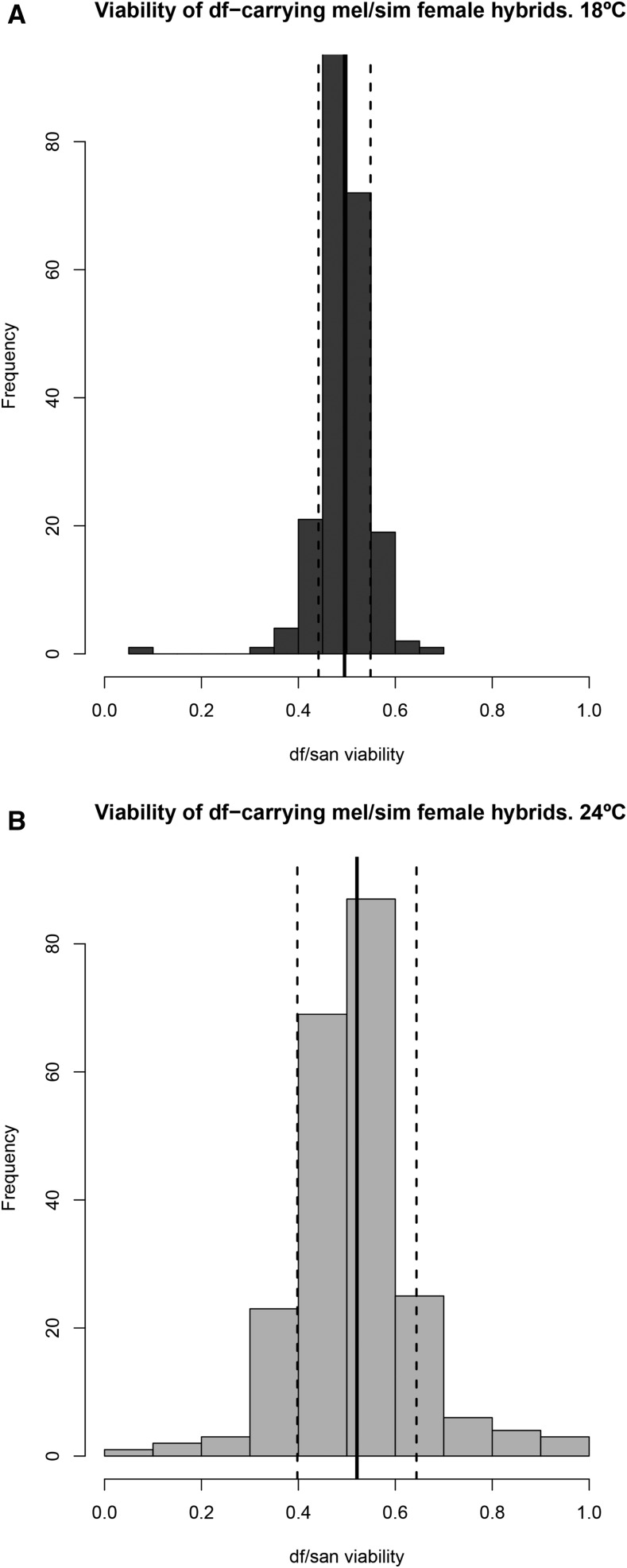
Fitness frequencies of the *df*-carrying hybrids in *mel/sim* hybrids at two different temperatures. (A) *mel/sim* 18°. (B) *mel/sim* 24°. Black solid lines in each panel show the mean fitness of the *df*-carrying hybrids. Black dashed lines show the average ± SD from the mean. The *x*-axis shows relative viability of deficiency-carrying progeny (observed *df*-carrying progeny/observed *Bal*-carrying progeny + observed *df*-carrying progeny) while the *y*-axis shows the number of stocks having a given level of viability of deficiency carrying offspring. *Bal*, balancer chromosome; *df*, deficiencies; *mel*, *D. melanogaster*; *sim*, *D. simulans*.

We next compared the average effect size of exposing recessive alleles in the *D. santomea* genome in *mel/san* hybrid females at the two temperatures. This constitutes a test for the effect of temperature on the penetrance of alleles involved in HI. First, we looked at the fitness distributions at the two temperatures for both hybrids: *mel/san* (Figure 3 and Figure 4) and *mel/sim* (Figure 5). Even though 69 of 125 individual loci cause HI at only one temperature in *mel/san* hybrids (Table S2, see above), the genome-wide effect of temperature on the fitness of *df*-carrying hybrids is modest and nonsignificant (mean difference between fitness at 18 and 24°= −0.0120; 95% C.I.: −0.0488, 0.0089; paired *t*-test; *t* = −1.3659, d.f. = 222, *P* = 0.1734). In *mel/sim* hybrids, we found that temperature has a strong effect on the fitness of *df*-carrying hybrids, and that these hybrids are more viable at 24° (mean difference between fitness at 18 and 24° = −0.0256; 95% C.I.: −0.0426, −0.0086; paired *t*-test; *t* = −2.968, d.f. = 222, *P* = 3.326 × 10^−4^).

We also fitted a linear model to jointly assess the relative contributions of paternal species and temperature. We found that the fitness distribution of *df*-carrying hybrids differs significantly between *mel/san* and *mel/sim* hybrids (Table 1, species effect). We also found that the magnitude of HI is not affected by the rearing temperature *per se* (Table 1, temperature effect), but it is affected by the interaction between the parental species and rearing temperature (Table 1, temperature × species interaction). To quantify the importance of the species, we fitted a linear model dependent only on the temperature × species interaction. We found that temperature affects inviability differently between species (*F*_3888_ = 16.018, *P* = 3.785 × 10^−10^). *df*-carrying *mel/sim* hybrids are more fit on average than *df*-carrying *mel/san* hybrids at 18° (linear contrasts with multiple comparison corrections: viability of *df/sim* − viability of *df/san* at 18°: estimate = 0.0415; *t* = 2.980; *P* = 0.0157). Similarly, *df*-carrying *mel/sim* hybrids are also more fit than *df*-carrying *mel/san* hybrids at 24° (viability of *df/sim* − viability of *df/san* at 24°: estimate = 0.0871; *t* = 6.252, *P* < 0.001).

**Table 1:** Levels of heterogeneity at relative viability of df(*i*)/(*j*) hybrids, where (*i*) represents a deficiency stock and (*j*) represents either of the two parental species

	Degrees of Freedom	Sum of Squares	Mean Square Error	*F* Value	Pr (> *F*)
Species	1	0.9226	0.9226	42.6207	1.116 × 10^−10^
Temperature	1	0.0018	0.0018	0.0818	0.7750
Temperature × species	1	0.1159	0.1159	5.3523	0.0209
Residuals	888	19.2219	0.0217		

Linear Model: HI ∼ Temp + Species + (Temperature × Species) shows that the two fixed effects (temperature and species of the father), and the interaction between these two effects determines the viability of hybrids. HI, hybrid inviability; Pr, .

#### Effect of chromosomal location:

We next explored whether temperature caused differences in the magnitude of HI between *X*-linked and autosomal regions in the two interspecific hybrids. We found that temperature-dependent viability is not contingent on chromosomal location and that temperature has similar effects on *X*-linked and autosomal alleles in both hybrids (chromosome × species × temperature interaction: *F*_2880_ = 2.3132, *P* = 0.0995). Given the large number of possible pairwise comparisons (66 comparisons, Table S4), we restricted our analyses to six comparisons, all within species and only accounting for the interaction term. Pairwise comparisons within species confirmed that the effect of temperature on the penetrance of HI alleles is minimal in both types of hybrids (*mel/sim* and *mel/san*), and that none of the three chromosomes is more prone to show differential HI when raised at different temperatures (Table 2, rows 4–6).

**Table 2:** Pairwise comparisons (Tukey HSD test) from a linear model show that in *mel/san* hybrids, only chromosome two is marginally affected by temperature, and the effect size is modest

Linear Hypothesis	Mean 1	Mean 2	Estimate	SE	*t* Value	P-value
*X*.*sim*.24° − *X*.sim.18° == 0	0.4975	0.4465	0.0402	0.0286	1.407	0.9612
2.*sim*.24° − 2.sim.18° == 0	0.5278	0.4964	0.0314	0.0206	1.524	0.9323
3.*sim*.24° − 3.*sim*.18° == 0	0.4986	0.4917	0.0068	0.0244	0.279	1.0000
*X*.*san*.24° − *X*.*san*.18° == 0	0.4014	0.5377	0.0451	0.0286	1.579	0.9144
2.*san*.24° − 2.*san*.18° == 0	0.4430	0.5027	−0.0598	0.0206	−2.901	0.1399
3.*san*.24° − 3.*san*.18° == 0	0.4113	0.4229	−0.0116	0.0244	−0.475	1.0000

The first column shows the pairwise comparisons (Chromosome.Species.Temperature). Mean 1 refers to the mean of the first category listed in the comparison; Mean 2 refers to the mean of the second category. The effect of temperature was not significant in either *mel/sim* or *mel/san* hybrids. P-value, ; *mel*, *D. melanogaster*; *san*, *D. santomea*; *sim*, *D. simulans*.

## Discussion

HI is one of the most extreme phenotypes of reproductive isolation and constitutes both an important barrier to gene flow and an important mechanism for completing speciation (Coyne and Orr 2004; Noor and Feder 2006; Edmands 2007). Although it has generally been considered to be more environmentally-independent than prezygotic isolation (Coyne and Orr 2004; Sobel *et al.* 2010), the penetrance of HI is affected by extrinsic factors such as temperature (Wade and Johnson 1994; Wade *et al.* 1999). In this report, we measured the penetrance of HI alleles in two interspecific *Drosophila* crosses at two different temperatures. While temperature has a stronger effect on the penetrance of hybrid incompatibility loci in *mel/sim* hybrids than in *mel/san* hybrids, the overall results from both crosses suggest that temperature plays an important role in HI. Consistent with previous findings (Matute *et al.* 2010; Matute and Gavin-Smyth 2014), we found that *mel/san* hybrids have many more incompatibilities than *mel/sim* hybrids, as expected based on their longer divergence time. We also found that most HI alleles in *mel/san* hybrids are deleterious at both temperatures. Our results strongly indicate that the penetrance of these incompatibilities is independent from temperature (at least at the two assessed temperatures). Yet, there are alleles that cause inviability only at 18° or only at 24°, indicating that postzygotic isolation in this cross can still be affected by extrinsic factors. In *mel/sim* hybrids we found the opposite pattern; the magnitude of HI is strongly dependent on the temperature at which the hybrids are raised.

Only one of the identified loci causes HI at both 18 and 24°, indicating that different sets of loci affect HI at different temperatures. Given these data, our initial hypothesis that *D. santomea*’s temperature specialization would cause *mel/san* hybrids to be more affected by temperature than *mel/sim* hybrids is unlikely to be correct. If temperature had a strong effect on hybrid incompatibilities in *mel/san* hybrids, we would expect to see far more temperature-dependent hybrid incompatibilities than temperature-independent hybrid incompatibilities, a pattern we do not observe.

When we evaluated the mean effect size of *D. santomea* recessive HI alleles in *mel/san* hybrids, we found that the mean viability of *df*-carrying hybrids is similar at 24° and at 18°, a somewhat surprising result. We expected that *mel/san* hybrids would be more temperature sensitive due to the narrow temperature range inhabited by *D. santomea* (Matute *et al.* 2009a; Comeault *et al.* 2016). The mean magnitude of HI in *mel/sim* hybrids, unlike the pattern observed in *mel/san* hybrids, is contingent on temperature, and *df*-carrying hybrids do better at 24° than at 18°. This result is surprising because, unlike *D. santomea*, *D. simulans* is a widely cosmopolitan species that is able to breed at a range of temperatures (Austin and Moehring 2013), and we expected that *D. simulans* hybrids would be less affected by temperature.

A possible explanation for this pattern is that hybrids between highly divergent species (*mel/san*) are less likely to be affected by temperature because their genomes contain a larger number of loci with potentially deleterious interactions (Orr 1995; Matute *et al.* 2010; Moyle and Nakazato 2010; Wang *et al.* 2015). Alternatively, increased divergence time between species likely leads to an increase in the number of loci involved in HI, which might be expected to lead to reduced temperature sensitivity. In such cases, the penetrance of hybrid incompatibilities might be less likely to be affected by environmental factors due to the very large number of deleterious interactions. Even with a moderate reduction in the number of interactions at a lower temperature, many other deleterious interactions will remain and cause HI. Conversely, more recently diverged species will have fewer deleterious interactions, and so may be more strongly affected by temperature as each single interaction plays a larger role in HI. It is also possible that *D. santomea*’s temperature specialization has resulted in lower variability among alleles involved in thermal preference/thermal tolerance. This may result in lower variability of outcomes between temperatures because each allele has similar fitness at each temperature.

Temperature-dependent HI alleles in *mel/sim* hybrids could hypothetically serve as an intermediate state for gene flow between populations, allowing successful production of progeny under only certain conditions. This is an unlikely explanation, however, as hybrids between *D. melanogaster* and *D. simulans* or *D. santomea* are inviable, or sterile, and have never been observed in nature.

Our results have one caveat. We cannot address whether the penetrance of alleles involved in hybrid incompatibility is more or less pronounced in interspecific hybrids from parents with a restricted thermal niche than in interspecific hybrids with a wide thermal niche. Our experiment does not allow us to disentangle the effects of genetic distance between hybrids and the identity of the examined species. An ideal test would involve comparing the penetrance of recessive HI alleles between pairs of hybrids whose parents have roughly equivalent genetic distances. To study highly divergent hybrids, one could study hybrids between *D. melanogaster* with *D. santomea* and between *D. melanogaster* with *D. yakuba*. Since *D. santomea* and *D. yakuba* are sister species, their levels of divergence from *D. melanogaster* are roughly equivalent (Turissini *et al.* 2016). However, multiple attempts to hybridize *D. melanogaster* and *D. yakuba* have failed and, when hybridization has succeeded, the protocol is onerous and unlikely to be applicable to a genome-wide mapping approach (Sanchez and Santamaria 1997). Another possibility is to compare the viability of *mel/san* hybrids with hybrids between *D. melanogaster* and *D. teissieri*, which is related to *D. santomea* and *D. yakuba*. A second set of potentially informative crosses would be *D. melanogaster* with *D. simulans* and *D. melanogaster* with *D. sechellia* (or *D. mauritiana*). The *D*. *simulans/sechellia/mauritiana* triad might also be useful to assess whether there are interactions between the mitochondrial and endosymbiont genomes, the nuclear genome, and the temperature at which the hybrids are raised.

Several studies have suggested that hybrid defects are more common and more severe at high temperatures (Wade and Johnson 1994, Wade *et al.* 1999, Bordenstein and Drapeau 2001). The genetic underpinnings of such interactions remain unknown, although potential explanations have included differences in molecular kinetics and a high correlation of thermal tolerance alleles with DMIs. Our finding that *df*-carrying *mel/sim* hybrids have higher overall viability at 24 than 18° is surprising. Previous work examining the interaction of temperature and hybrid viability has found that hybrid viability decreases at higher temperatures (Koevoets *et al.* 2012), disagreeing with our finding. In the case of *df*-carrying *mel/san* hybrids, the influence of temperature on viability was negligible. This suggests that, at the very least, the interaction of temperature and HI alleles is complex and likely varies depending on the species pair.

One possible explanation for this unexpected result is temperature-dependent haploinsufficiency. *df*-carrying hybrids have only a single copy of each gene located within the particular deficiency they carry. Though these regions are known not to cause haploinsufficiency when hemizygous in the parental species, it is unknown if these regions will be haploinsufficient in *mel/sim* hybrids but not in *mel/san* hybrids. The single copy of the gene product at these loci may be sufficient in the hybrids when reared at 24° but suffer too great a loss of function and become insufficient due to the reduced kinetics at 18°. These loci may become haploinsufficient in the hybrids at the lower temperature due to reduced function of the gene product at 18°. In this case, haploinsufficiency would be contingent on genetic background (*i.e.*, the identity of the hybrid), suggesting species-specific epistatic interactions and not generalized haploinsufficiency. It is also possible that these regions harbor temperature-dependent recessive lethal alleles segregating naturally in *D. simulans*, but not *D. santomea*, although this is an unlikely explanation for the observed pattern, as both species are capable of breeding at 18° (Matute *et al.* 2009b; Austin and Moehring 2013). If 18°-dependent recessive lethal variants segregated naturally in these species at frequencies high enough to be detected by our mapping, we would expect to see substantial reductions in fitness in these species when reared at 18°.

Our results show that temperature can play a significant role in the penetrance of HI, and that the effect of temperature varies depending on the species pair. Overall, our results and those from similar reports suggest that we should not think of HI solely as the product of genetic interactions in the hybrid offspring, but rather must consider the phenomenon of HI within the broader environmental and organismal context in which it is observed.

## Supplementary Material

Supplemental material is available online at www.g3journal.org/lookup/suppl/doi:10.1534/g3.116.034926/-/DC1.
