# Supplementary material for: The Effect of Temperature on Drosophila Hybrid Fitness
Source: G3 (Bethesda). 2016 Dec 2;7(2):377–85. doi: 10.1534/g3.116.034926 (PMC5295587; doi:10.1534/g3.116.034926)
Supplement: Supplementary file 1 [file 377TableS1.docx]

**TABLE S1**. Mutant strains used in this report. The 'Stock' column refers to the number of the mutant in Flybase. The table also includes all the raw data (counts) for the two types of interspecific crosses.

| **Stock** | **cytology** | ***Bal/san* progeny at 18ºC** | ***df/san* progeny at 18ºC** | **Proportion *df/san* 18ºC** | ***Bal/sim* progeny at 18ºC** | ***df/sim* progeny at 18ºC** | **Proportion *df/sim* 18ºC** |
| --- | --- | --- | --- | --- | --- | --- | --- |
| **1329** | 1A1--2A | 76 | 31 | 0.289719626 | 98 | 104 | 0.514851485 |
| **25058** | 1A5--1B12 | 37 | 0 | 0 | 78 | 104 | 0.571428571 |
| **25062** | 1D1--2A3 | 206 | 115 | 0.358255452 | 134 | 156 | 0.537931034 |
| **9054** | 2E1--3A2 | 107 | 153 | 0.588461538 | 204 | 231 | 0.531034483 |
| **26569** | 2F2--3A4 | 181 | 325 | 0.64229249 | 55 | 47 | 0.460784314 |
| **935** | 2F6--3C5 | 266 | 270 | 0.504672897 | 29 | 21 | 0.42 |
| **8031** | 3A3--3A8 | 101 | 160 | 0.61302682 | 174 | 199 | 0.533512064 |
| **9348** | 3A8--3B1 | 85 | 65 | 0.433333333 | 205 | 234 | 0.533029613 |
| **8948** | 3B1--3C5 | 145 | 0 | 0 | 99 | 111 | 0.528571429 |
| **939** | 3C11--3E4 | 250 | 180 | 0.418604651 | 104 | 131 | 0.557446809 |
| **729** | 3C1--3D6 | 240 | 105 | 0.304347826 | 209 | 190 | 0.476190476 |
| **944** | 4C15--5A2 | 65 | 55 | 0.458333333 | 202 | 188 | 0.482051282 |
| **945** | 5A8--5C6 | 180 | 170 | 0.485714286 | 196 | 171 | 0.465940054 |
| **26506** | 5B6--5D2 | 104 | 48 | 0.315789474 | 174 | 156 | 0.472727273 |
| **946** | 5C2--5D6 | 168 | 151 | 0.473354232 | 97 | 78 | 0.445714286 |
| **8947** | 5C7--5F3 | 108 | 63 | 0.368421053 | 154 | 178 | 0.536144578 |
| **7713** | 5F2--6B2 | 347 | 331 | 0.48820059 | 185 | 199 | 0.518229167 |
| **7714** | 6B2--6C4 | 70 | 71 | 0.503546099 | 167 | 200 | 0.544959128 |
| **23670** | 6C11--6D3 | 56 | 41 | 0.422680412 | 172 | 142 | 0.452229299 |
| **9625** | 6C12--6D8 | 155 | 185 | 0.544117647 | 132 | 111 | 0.456790123 |
| **25063** | 6C2--6C8 | 80 | 50 | 0.384615385 | 145 | 167 | 0.53525641 |
| **3196** | 6E2--7A6 | 330 | 325 | 0.496183206 | 65 | 82 | 0.557823129 |
| **947** | 6E4--7A6 | 135 | 145 | 0.517857143 | 198 | 206 | 0.50990099 |
| **8955** | 7A3--7B2 | 220 | 221 | 0.501133787 | 200 | 225 | 0.529411765 |
| **3221** | 7B2-7C4 | 55 | 50 | 0.476190476 | 75 | 91 | 0.548192771 |
| **949** | 7D1--7D6 | 56 | 0 | 0 | 205 | 183 | 0.471649485 |
| **950** | 7D10--8A5 | 159 | 115 | 0.419708029 | 165 | 195 | 0.541666667 |
| **951** | 7F1--8C6 | 44 | 0 | 0 | 145 | 152 | 0.511784512 |
| **3651** | 8B5--8D9 | 121 | 96 | 0.442396313 | 97 | 109 | 0.529126214 |
| **952** | 8E--9D | 543 | 314 | 0.366394399 | 142 | 104 | 0.422764228 |
| **954** | 9B1-2;10A1-2 | 81 | 95 | 0.539772727 | 185 | 205 | 0.525641026 |
| **7339** | 9D5-9E8 | 492 | 369 | 0.428571429 | 164 | 199 | 0.548209366 |
| **5707** | 9E3--10A8 | 467 | 414 | 0.469920545 | 79 | 96 | 0.548571429 |
| **26556** | 9E4--9F12 | 61 | 77 | 0.557971014 | 561 | 501 | 0.471751412 |
| **25068** | 9E8--10A3 | 274 | 179 | 0.395143488 | 311 | 267 | 0.461937716 |
| **962** | 10F7--11D1 | 96 | 49 | 0.337931034 | 253 | 271 | 0.517175573 |
| **967** | 11D--12A2 | 272 | 448 | 0.622222222 | 274 | 290 | 0.514184397 |
| **727** | 12A3--12E9 | 34 | 102 | 0.75 | 185 | 177 | 0.488950276 |
| **998** | 12D2--13A5 | 12 | 14 | 0.538461538 | 184 | 184 | 0.5 |
| **3347** | 13F1--14B1 | 69 | 105 | 0.603448276 | 105 | 98 | 0.482758621 |
| **125** | 14B8--14C1 | 145 | 133 | 0.478417266 | 57 | 78 | 0.577777778 |
| **991** | 14F6--15A6 | 172 | 254 | 0.596244131 | 289 | 251 | 0.464814815 |
| **25416** | 15A1--15E2 | 193 | 133 | 0.40797546 | 161 | 145 | 0.473856209 |
| **4741** | 15D3--16A6 | 331 | 288 | 0.465266559 | 400 | 310 | 0.436619718 |
| **4953** | 16A2--16C10 | 306 | 271 | 0.469670711 | 203 | 172 | 0.458666667 |
| **970** | 17A1--18A2 | 77 | 61 | 0.442028986 | 48 | 32 | 0.4 |
| **7754** | 18A2--18A3 | 64 | 107 | 0.625730994 | 262 | 251 | 0.489278752 |
| **971** | 18A5--18D | 70 | 0 | 0 | 145 | 131 | 0.474637681 |
| **7721** | 18D13--18F2 | 117 | 46 | 0.282208589 | 172 | 145 | 0.457413249 |
| **972** | 18E1--20F | 88 | 1 | 0.011235955 | 423 | 425 | 0.501179245 |
| **977** | 19F1--20F | 156 | 1 | 0.006369427 | 461 | 515 | 0.527663934 |
| **3714** | 20A--20F | 76 | 2 | 0.025641026 | 304 | 165 | 0.351812367 |
| **3638** | 21A1--21B8 | 247 | 241 | 0.493852459 | 298 | 256 | 0.462093863 |
| **8672** | 21B7--21C2 | 244 | 247 | 0.50305499 | 318 | 341 | 0.517450683 |
| **6283** | 21B7--21C3 | 143 | 133 | 0.481884058 | 68 | 79 | 0.537414966 |
| **6608** | 21C3--21C8 | 36 | 23 | 0.389830508 | 451 | 402 | 0.471277843 |
| **3084** | 21D1--22B3 | 161 | 106 | 0.397003745 | 382 | 311 | 0.448773449 |
| **24120** | 22B2--22D4 | 227 | 166 | 0.422391858 | 288 | 144 | 0.333333333 |
| **7144** | 22D2--22F2 | 139 | 222 | 0.614958449 | 167 | 155 | 0.48136646 |
| **90** | 22F4--23C4 | 111 | 108 | 0.493150685 | 211 | 208 | 0.496420048 |
| **1567** | 23C1--23E2 | 71 | 128 | 0.64321608 | 49 | 61 | 0.554545455 |
| **6875** | 23C5--23E2 | 661 | 721 | 0.52170767 | 178 | 156 | 0.467065868 |
| **6965** | 23E5--23F5 | 151 | 176 | 0.5382263 | 291 | 283 | 0.493031359 |
| **6507** | 23F3--24A2 | 94 | 105 | 0.527638191 | 105 | 100 | 0.487804878 |
| **5330** | 24A2--24D4 | 104 | 93 | 0.472081218 | 146 | 101 | 0.408906883 |
| **693** | 24C2--25C9 | 191 | 313 | 0.621031746 | 56 | 45 | 0.445544554 |
| **9270** | 24F4--25A7 | 271 | 316 | 0.538330494 | 94 | 111 | 0.541463415 |
| **8835** | 25C1--25C4 | 456 | 311 | 0.40547588 | 299 | 282 | 0.485370052 |
| **8674** | 25C4--25C8 | 329 | 287 | 0.465909091 | 415 | 471 | 0.531602709 |
| **7497** | 25C8--25D5 | 163 | 168 | 0.50755287 | 261 | 243 | 0.482142857 |
| **781** | 25D2--26B5 | 251 | 438 | 0.635703919 | 149 | 167 | 0.528481013 |
| **490** | 25F3--26D11 | 116 | 111 | 0.488986784 | 45 | 54 | 0.545454545 |
| **6299** | 26B1--26D2 | 487 | 496 | 0.504577823 | 167 | 201 | 0.546195652 |
| **6374** | 26D10--27C1 | 179 | 197 | 0.52393617 | 188 | 165 | 0.467422096 |
| **2414** | 27C1--28A | 131 | 398 | 0.752362949 | 199 | 183 | 0.479057592 |
| **5420** | 27C2--27C5 | 101 | 113 | 0.528037383 | 74 | 99 | 0.572254335 |
| **4956** | 27E2--28D1 | 145 | 356 | 0.710578842 | 153 | 203 | 0.570224719 |
| **7147** | 28A4--28D9 | 146 | 165 | 0.530546624 | 205 | 204 | 0.498777506 |
| **140** | 28D2--28E5 | 301 | 657 | 0.685803758 | 106 | 121 | 0.533039648 |
| **179** | 28E4--29C1 | 62 | 88 | 0.586666667 | 413 | 231 | 0.358695652 |
| **8836** | 28F5--29B1 | 141 | 213 | 0.601694915 | 197 | 209 | 0.514778325 |
| **9298** | 29B4--29C3 | 171 | 146 | 0.460567823 | 164 | 151 | 0.479365079 |
| **2892** | 29C1--30C9 | 202 | 241 | 0.544018059 | 205 | 231 | 0.529816514 |
| **6478** | 30C3--30F1 | 184 | 187 | 0.504043127 | 95 | 76 | 0.444444444 |
| **1045** | 30D--31F | 138 | 153 | 0.525773196 | 341 | 267 | 0.439144737 |
| **8469** | 30F5--31B1 | 701 | 318 | 0.312070658 | 410 | 541 | 0.568874869 |
| **3366** | 31B1--32A2 | 166 | 111 | 0.400722022 | 51 | 66 | 0.564102564 |
| **9503** | 31B1--31D9 | 201 | 231 | 0.534722222 | 67 | 81 | 0.547297297 |
| **7142** | 32A1--32D1 | 114 | 92 | 0.446601942 | 188 | 154 | 0.450292398 |
| **9505** | 32C1--32C1 | 245 | 413 | 0.627659574 | 212 | 200 | 0.485436893 |
| **7143** | 32D1--32E1 | 331 | 528 | 0.614668219 | 431 | 398 | 0.480096502 |
| **5869** | 32D1--32F3 | 186 | 306 | 0.62195122 | 291 | 187 | 0.391213389 |
| **3079** | 32F1--33F2 | 72 | 51 | 0.414634146 | 174 | 200 | 0.534759358 |
| **6999** | 34A3--34B9 | 539 | 207 | 0.277479893 | 560 | 501 | 0.472196041 |
| **9594** | 34B4--34C4 | 166 | 118 | 0.415492958 | 18 | 31 | 0.632653061 |
| **9506** | 34C1--34C6 | 171 | 153 | 0.472222222 | 24 | 24 | 0.5 |
| **3588** | 35B4--35F7 | 46 | 0 | 0 | 67 | 66 | 0.496240602 |
| **1491** | 35D1--36A7 | 192 | 221 | 0.535108959 | 204 | 201 | 0.496296296 |
| **420** | 36C2--37C1 | 34 | 27 | 0.442622951 | 56 | 52 | 0.481481481 |
| **567** | 37B2--38D5 | 183 | 123 | 0.401960784 | 56 | 67 | 0.544715447 |
| **167** | 38A6--40B1 | 96 | 48 | 0.333333333 | 186 | 191 | 0.5066313 |
| **7531** | 40A5--40D3 | 321 | 451 | 0.584196891 | 203 | 201 | 0.497524752 |
| **9510** | 40A5--40E5 | 172 | 11 | 0.06010929 | 205 | 191 | 0.482323232 |
| **4959** | h35--40A1 | 124 | 7 | 0.053435115 | 56 | 67 | 0.544715447 |
| **749** | h44--42A2 | 103 | 58 | 0.360248447 | 41 | 51 | 0.554347826 |
| **1888** | 42B3--43E18 | 81 | 58 | 0.417266187 | 34 | 23 | 0.403508772 |
| **3368** | 42E--44C | 1 | 21 | 0.954545455 | 178 | 209 | 0.54005168 |
| **198** | 43F--44D8 | 141 | 116 | 0.451361868 | 94 | 100 | 0.515463918 |
| **201** | 44D1--44F12 | 99 | 87 | 0.467741935 | 204 | 183 | 0.472868217 |
| **3591** | 44F10--45E1 | 203 | 198 | 0.493765586 | 202 | 204 | 0.502463054 |
| **4966** | 45A6--45E3 | 56 | 65 | 0.537190083 | 147 | 165 | 0.528846154 |
| **6917** | 45D3--45F6 | 190 | 211 | 0.526184539 | 512 | 493 | 0.490547264 |
| **9410** | 45F6--46B4 | 177 | 71 | 0.286290323 | 204 | 216 | 0.514285714 |
| **1743** | 46A--46C | 129 | 62 | 0.32460733 | 22 | 19 | 0.463414634 |
| **1702** | 46C--47A1 | 71 | 0 | 0 | 104 | 86 | 0.452631579 |
| **190** | 47D3--48B2 | 266 | 123 | 0.316195373 | 205 | 174 | 0.459102902 |
| **1145** | 48A3--48C8 | 56 | 116 | 0.674418605 | 319 | 301 | 0.485483871 |
| **7145** | 48C5--48E1 | 145 | 121 | 0.454887218 | 238 | 198 | 0.45412844 |
| **7146** | 48E1--48E10 | 154 | 478 | 0.756329114 | 58 | 56 | 0.49122807 |
| **5879** | 48E12--49B6 | 48 | 36 | 0.428571429 | 194 | 199 | 0.506361323 |
| **754** | 49B2--49E2 | 71 | 48 | 0.403361345 | 204 | 203 | 0.498771499 |
| **442** | 49C1--50D1 | 116 | 101 | 0.465437788 | 198 | 402 | 0.67 |
| **6516** | 50D1--50D7 | 92 | 116 | 0.557692308 | 311 | 341 | 0.523006135 |
| **7875** | 50D4--50E4 | 24 | 32 | 0.571428571 | 287 | 302 | 0.512733447 |
| **9496** | 50E1--50E6 | 73 | 58 | 0.442748092 | 195 | 211 | 0.519704433 |
| **7876** | 50E4--50F6 | 136 | 118 | 0.464566929 | 174 | 209 | 0.545691906 |
| **6455** | 50E6--51E4 | 156 | 1 | 0.006369427 | 174 | 151 | 0.464615385 |
| **3518** | 51D3-52F9 | 7 | 176 | 0.961748634 | 188 | 154 | 0.450292398 |
| **3520** | 52F5--53A1 | 44 | 45 | 0.505617978 | 173 | 142 | 0.450793651 |
| **25078** | 53C1--53C6 | 248 | 421 | 0.629297459 | 47 | 54 | 0.534653465 |
| **7445** | 53D9--54B10 | 61 | 41 | 0.401960784 | 69 | 67 | 0.492647059 |
| **7414** | 54B1--54B10 | 59 | 201 | 0.773076923 | 419 | 411 | 0.495180723 |
| **5574** | 54B16--54B16 | 245 | 321 | 0.567137809 | 242 | 230 | 0.487288136 |
| **5680** | 54B17--C4 | 211 | 217 | 0.507009346 | 39 | 42 | 0.518518519 |
| **9596** | 54B2--54B17 | 82 | 78 | 0.4875 | 211 | 246 | 0.538293217 |
| **6780** | 54E5--55B7 | 236 | 463 | 0.662374821 | 252 | 211 | 0.455723542 |
| **1547** | 55A--55F | 41 | 36 | 0.467532468 | 241 | 267 | 0.525590551 |
| **757** | 55E2--56C11 | 27 | 256 | 0.90459364 | 71 | 78 | 0.523489933 |
| **6866** | 56C4--56D10 | 133 | 643 | 0.828608247 | 221 | 211 | 0.488425926 |
| **6647** | 56D7--56F12 | 67 | 58 | 0.464 | 189 | 195 | 0.5078125 |
| **7896** | 56F11--56F16 | 176 | 223 | 0.558897243 | 167 | 145 | 0.46474359 |
| **3467** | 56F9--57D12 | 71 | 1 | 0.013888889 | 154 | 141 | 0.477966102 |
| **5246** | 57D2--58D1 | 166 | 34 | 0.17 | 154 | 180 | 0.538922156 |
| **282** | 58D1--59A | 29 | 262 | 0.900343643 | 41 | 32 | 0.438356164 |
| **3909** | 59A1--59D4 | 258 | 273 | 0.514124294 | 56 | 78 | 0.582089552 |
| **7273** | 59B--59E1 | 121 | 398 | 0.766859345 | 78 | 75 | 0.490196078 |
| **1682** | 59D5--60B8 | 11 | 91 | 0.892156863 | 104 | 111 | 0.51627907 |
| **9691** | 60B8--60C4 | 122 | 461 | 0.790737564 | 57 | 45 | 0.441176471 |
| **2604** | 60C5--60D10 | 78 | 91 | 0.538461538 | 82 | 73 | 0.470967742 |
| **9069** | 60C8--60E8 | 123 | 31 | 0.201298701 | 41 | 30 | 0.422535211 |
| **2471** | 60E2--60E12 | 76 | 58 | 0.432835821 | 194 | 160 | 0.451977401 |
| **4961** | 60F1--60F5 | 19 | 76 | 0.8 | 120 | 111 | 0.480519481 |
| **2577** | 61A--61D3 | 162 | 161 | 0.498452012 | 314 | 301 | 0.489430894 |
| **439** | 61C5--62A8 | 129 | 102 | 0.441558442 | 151 | 132 | 0.466431095 |
| **600** | 62A10--62D5 | 108 | 103 | 0.488151659 | 242 | 231 | 0.488372093 |
| **9693** | 62A11--62B7 | 104 | 38 | 0.267605634 | 101 | 89 | 0.468421053 |
| **2400** | 62B4--62E5 | 111 | 86 | 0.436548223 | 103 | 74 | 0.418079096 |
| **6755** | 62E8--63B6 | 47 | 1 | 0.020833333 | 204 | 151 | 0.425352113 |
| **3650** | 62F--63B10 | 179 | 162 | 0.475073314 | 138 | 135 | 0.494505495 |
| **3649** | 63C2--63F7 | 67 | 89 | 0.570512821 | 99 | 138 | 0.582278481 |
| **463** | 63E6--64A10 | 75 | 108 | 0.590163934 | 56 | 71 | 0.559055118 |
| **3686** | 63F6--64C15 | 92 | 92 | 0.5 | 104 | 92 | 0.469387755 |
| **3096** | 64C--65C | 40 | 52 | 0.565217391 | 156 | 204 | 0.566666667 |
| **4393** | 65A2;65E1 | 133 | 90 | 0.403587444 | 204 | 200 | 0.495049505 |
| **6867** | 65D4--65E6 | 100 | 92 | 0.479166667 | 89 | 142 | 0.614718615 |
| **6964** | 65E10--65F6 | 236 | 192 | 0.448598131 | 203 | 190 | 0.48346056 |
| **1420** | 65F3;66B10 | 160 | 91 | 0.362549801 | 138 | 165 | 0.544554455 |
| **5877** | 66A17-20;66C1-5 | 55 | 80 | 0.592592593 | 104 | 11 | 0.095652174 |
| **6460** | 66B12-C1;66D2-4 | 49 | 55 | 0.528846154 | 202 | 198 | 0.495 |
| **1541** | 66B8-9;66C9-10 | 194 | 170 | 0.467032967 | 243 | 202 | 0.453932584 |
| **3024** | 66D10--66E2 | 51 | 0 | 0 | 105 | 111 | 0.513888889 |
| **4500** | 66E1-6;66F1-6 | 55 | 45 | 0.45 | 67 | 87 | 0.564935065 |
| **7079** | 66F1-2;67B2-3 | 92 | 102 | 0.525773196 | 88 | 96 | 0.52173913 |
| **997** | 67A2;67D7-13 or 67A5;67D9-13 | 41 | 52 | 0.559139785 | 92 | 79 | 0.461988304 |
| **23668** | 67C7;67D5 | 21 | 33 | 0.611111111 | 96 | 90 | 0.483870968 |
| **2612** | 68C8--69B5 | 115 | 40 | 0.258064516 | 92 | 103 | 0.528205128 |
| **5492** | 69A4--69D6 | 120 | 99 | 0.452054795 | 104 | 88 | 0.458333333 |
| **6456** | 69D4-5;69F5-7 | 52 | 42 | 0.446808511 | 205 | 194 | 0.486215539 |
| **3124** | 70C1-2;70D4-5 | 320 | 288 | 0.473684211 | 99 | 78 | 0.440677966 |
| **3126** | 70D2-3;71E4-5 | 216 | 311 | 0.590132827 | 104 | 100 | 0.490196078 |
| **6551** | 71C2-3;72B1-C1 | 57 | 82 | 0.589928058 | 203 | 222 | 0.522352941 |
| **2993** | 72C1-D1;73A3-4 | 154 | 98 | 0.388888889 | 311 | 278 | 0.471986418 |
| **6411** | 74D3-75A1;75B2-5 | 136 | 112 | 0.451612903 | 75 | 100 | 0.571428571 |
| **2608** | 75A6-7;75C1-2 | 36 | 21 | 0.368421053 | 84 | 92 | 0.522727273 |
| **6754** | 75F10--76A5 | 89 | 181 | 0.67037037 | 99 | 99 | 0.5 |
| **8082** | 75F2;76A1 | 124 | 133 | 0.517509728 | 75 | 77 | 0.506578947 |
| **3617** | 76B1-2;76D5 | 84 | 72 | 0.461538462 | 300 | 277 | 0.480069324 |
| **5126** | 76B4;77B | 36 | 0 | 0 | 218 | 211 | 0.491841492 |
| **2052** | 77A1;77D1 | 33 | 0 | 0 | 123 | 141 | 0.534090909 |
| **3127** | 77B-C;77F-78A | 97 | 101 | 0.51010101 | 35 | 51 | 0.593023256 |
| **4429** | 77F3;78C8-9 | 78 | 89 | 0.532934132 | 78 | 98 | 0.556818182 |
| **4430** | 78C5--79A1 | 211 | 180 | 0.460358056 | 402 | 378 | 0.484615385 |
| **1990** | 83C1--84B2 | 136 | 79 | 0.36744186 | 250 | 222 | 0.470338983 |
| **1962** | 85A2;85C1-2 | 64 | 51 | 0.443478261 | 39 | 54 | 0.580645161 |
| **1931** | 85D8-12;85E7-F1 | 4 | 0 | 0 | 85 | 77 | 0.475308642 |
| **7080** | 85F1-2;86C7-8 | 476 | 64 | 0.118518519 | 84 | 98 | 0.538461538 |
| **3128** | 86C1;87B1-5 | 264 | 172 | 0.394495413 | 101 | 89 | 0.468421053 |
| **3003** | 86E2-4;87C6-7 | 124 | 12 | 0.088235294 | 203 | 199 | 0.495024876 |
| **383** | 88E7-13;89A1 | 128 | 116 | 0.475409836 | 241 | 200 | 0.453514739 |
| **756** | 88F9-89A1;89B9-10 | 63 | 34 | 0.350515464 | 142 | 111 | 0.438735178 |
| **1920** | 89B5;89C | 171 | 150 | 0.46728972 | 203 | 189 | 0.482142857 |
| **1467** | 89B7--89E7 | 29 | 41 | 0.585714286 | 222 | 259 | 0.538461538 |
| **4431** | 89E--91B2 | 85 | 0 | 0 | 89 | 99 | 0.526595745 |
| **3011** | 90F1-F4;91F5 | 49 | 64 | 0.566371681 | 54 | 42 | 0.4375 |
| **3012** | 91F1-2;92D3-6 | 206 | 171 | 0.453580902 | 99 | 111 | 0.528571429 |
| **4962** | 92B3;92F13 | 271 | 160 | 0.371229698 | 104 | 100 | 0.490196078 |
| **7413** | 92F7-93A1;93B3-6 | 248 | 151 | 0.378446115 | 204 | 191 | 0.483544304 |
| **26529** | 93D1;93F14 | 0 | 2 | 1 | 220 | 199 | 0.474940334 |
| **2586** | 94A3-4;94D1-4 | 28 | 20 | 0.416666667 | 231 | 241 | 0.51059322 |
| **7674** | 95A4;95B1 | 199 | 107 | 0.349673203 | 142 | 161 | 0.531353135 |
| **2585** | 95A5-7;95D6-11 | 331 | 201 | 0.377819549 | 200 | 188 | 0.484536082 |
| **4432** | 95D7-D11;95F15 | 75 | 107 | 0.587912088 | 312 | 299 | 0.489361702 |
| **2363** | 95F7--96A18 | 270 | 255 | 0.485714286 | 86 | 100 | 0.537634409 |
| **3468** | 96A2-7;96D2-4 | 127 | 121 | 0.487903226 | 93 | 87 | 0.483333333 |
| **5601** | 96F1;97B1 | 292 | 311 | 0.515754561 | 111 | 89 | 0.445 |
| **1910** | 97A--98A2 | 196 | 227 | 0.536643026 | 142 | 160 | 0.529801325 |
| **823** | 97E3--98A5 | 41 | 0 | 0 | 203 | 187 | 0.479487179 |
| **9529** | 97F1-2;98A | 48 | 43 | 0.472527473 | 163 | 142 | 0.46557377 |
| **7412** | 98B1-2;98B3-5 | 181 | 223 | 0.551980198 | 153 | 138 | 0.474226804 |
| **430** | 98E3;99A6-8 | 133 | 151 | 0.531690141 | 142 | 111 | 0.438735178 |
| **669** | 99A1-2;99B6-11 | 55 | 79 | 0.589552239 | 89 | 74 | 0.45398773 |
| **3547** | 99B5-6;99E4-F1, 98F;100F | 56 | 48 | 0.461538462 | 94 | 83 | 0.468926554 |
| **3546** | 99C8;100F5 | 96 | 16 | 0.142857143 | 102 | 81 | 0.442622951 |
