# Supplementary material for: The Effect of Temperature on Drosophila Hybrid Fitness
Source: G3 (Bethesda). 2016 Dec 2;7(2):377–85. doi: 10.1534/g3.116.034926 (PMC5295587; doi:10.1534/g3.116.034926)
Supplement: Supplementary file 2 [file 377TableS2.docx]

**TABLE S2.** Genome distribution of recessive *Drosophila santomea* hybrid incompatibilities in *mel/san* hybrids. We only scored the effect of deficiencies in five major Muller elements and excluded the dot-chromosome.

| Chromosome arm | Total number of deficiencies | Lethal deficiencies 24ºC only | Hybrid inviability regions 24ºC | Lethal deficiencies 18ºC only | Hybrid inviability regions 18ºC | Lethal deficiencies at both temperatures | Hybrid inviability regions at both temperatures |
| --- | --- | --- | --- | --- | --- | --- | --- |
| X | 28 | 2 | 2 | 10 | 8 | 16 | 11 |
| 2L | 32 | 9 | 9 | 11 | 8 | 12 | 8 |
| 2R | 27 | 10 | 8 | 6 | 5 | 11 | 10 |
| 3L | 19 | 6 | 5 | 6 | 6 | 7 | 6 |
| 3R | 19 | 7 | 7 | 2 | 2 | 10 | 8 |
| Total | 125 | 34 | 31 | 35 | 29 | 56 | 43 |
