# Supplementary material for: The Effect of Temperature on Drosophila Hybrid Fitness
Source: G3 (Bethesda). 2016 Dec 2;7(2):377–85. doi: 10.1534/g3.116.034926 (PMC5295587; doi:10.1534/g3.116.034926)
Supplement: Supplementary file 3 [file 377TableS3.docx]

**TABLE S3.** Distribution of recessive *Drosophila simulans* hybrid incompatibilities in *mel/sim* hybrids. We only scored the effect of deficiencies in five major Muller elements and excluded the dot-chromosome.

| Chromosome arm | Total number of deficiencies | Lethal deficiencies 24ºC only | Hybrid inviability regions 24ºC | Lethal deficiencies 18ºC only | Hybrid inviability regions 18ºC | Lethal deficiencies at both temperatures | Hybrid inviability regions at both temperatures |
| --- | --- | --- | --- | --- | --- | --- | --- |
| *X* | 6 | 2 | 2 | 3 | 2 | 1 | 1 |
| *2L* | 5 | 5 | 5 | 0 | 0 | 0 | 0 |
| *2R* | 0 | 0 | 0 | 0 | 0 | 0 | 0 |
| *3L* | 6 | 3 | 3 | 3 | 3 | 0 | 0 |
| *3R* | 6 | 6 | 4 | 0 | 0 | 0 | 0 |
| Total | 23 | 16 | 14 | 6 | 5 | 1 | 1 |
