# Supplementary material for: The Effect of Temperature on Drosophila Hybrid Fitness
Source: G3 (Bethesda). 2016 Dec 2;7(2):377–85. doi: 10.1534/g3.116.034926 (PMC5295587; doi:10.1534/g3.116.034926)
Supplement: Supplementary file 4 [file 377TableS4.docx]

**TABLE S4.** Pairwise comparisons of relative viability of deficiency stocks at different temperatures in *mel/sim* and *mel/san* hybrid F1 females. Bolded cells show pairwise comparisons also shown in Table 2.

| **Linear hypotheses** | **Estimate** | **Std. Error** | **t value** | **Pr(>\|t\|)** |
| --- | --- | --- | --- | --- |
| sim.18.2 - san.18.2 == 0 | -0.006276 | 0.020595 | -0.305 | 1 |
| **san.24.2 - san.18.2 == 0** | **-0.059755** | **0.020595** | **-2.901** | **0.1399** |
| sim.24.2 - san.18.2 == 0 | 0.025103 | 0.020595 | 1.219 | 0.987 |
| san.18.3 - san.18.2 == 0 | -0.079837 | 0.0226 | -3.533 | 0.0217 |
| sim.18.3 - san.18.2 == 0 | -0.010969 | 0.0226 | -0.485 | 1 |
| san.24.3 - san.18.2 == 0 | -0.091446 | 0.0226 | -4.046 | <0.01 |
| sim.24.3 - san.18.2 == 0 | -0.004148 | 0.0226 | -0.184 | 1 |
| san.18.*X* - san.18.2 == 0 | -0.101332 | 0.024898 | -4.07 | <0.01 |
| sim.18.*X* - san.18.2 == 0 | -0.005208 | 0.024898 | -0.209 | 1 |
| san.24.*X* - san.18.2 == 0 | -0.056232 | 0.024898 | -2.258 | 0.5015 |
| sim.24.*X* - san.18.2 == 0 | 0.034969 | 0.024898 | 1.404 | 0.9616 |
| san.24.2 - sim.18.2 == 0 | -0.053479 | 0.020595 | -2.597 | 0.2778 |
| **sim.24.2 - sim.18.2 == 0** | **0.031378** | **0.020595** | **1.524** | **0.9323** |
| san.18.3 - sim.18.2 == 0 | -0.073561 | 0.0226 | -3.255 | 0.0525 |
| sim.18.3 - sim.18.2 == 0 | -0.004694 | 0.0226 | -0.208 | 1 |
| san.24.3 - sim.18.2 == 0 | -0.085171 | 0.0226 | -3.769 | <0.01 |
| sim.24.3 - sim.18.2 == 0 | 0.002128 | 0.0226 | 0.094 | 1 |
| san.18.*X* - sim.18.2 == 0 | -0.095056 | 0.024898 | -3.818 | <0.01 |
| sim.18.*X* - sim.18.2 == 0 | 0.001068 | 0.024898 | 0.043 | 1 |
| san.24.*X* - sim.18.2 == 0 | -0.049957 | 0.024898 | -2.006 | 0.6844 |
| sim.24.*X* - sim.18.2 == 0 | 0.041244 | 0.024898 | 1.657 | 0.8849 |
| sim.24.2 - san.24.2 == 0 | 0.084857 | 0.020595 | 4.12 | <0.01 |
| san.18.3 - san.24.2 == 0 | -0.020082 | 0.0226 | -0.889 | 0.9992 |
| sim.18.3 - san.24.2 == 0 | 0.048786 | 0.0226 | 2.159 | 0.5755 |
| san.24.3 - san.24.2 == 0 | -0.031692 | 0.0226 | -1.402 | 0.962 |
| sim.24.3 - san.24.2 == 0 | 0.055607 | 0.0226 | 2.46 | 0.3618 |
| san.18.*X* - san.24.2 == 0 | -0.041577 | 0.024898 | -1.67 | 0.879 |
| sim.18.*X* - san.24.2 == 0 | 0.054547 | 0.024898 | 2.191 | 0.552 |
| san.24.*X* - san.24.2 == 0 | 0.003522 | 0.024898 | 0.141 | 1 |
| sim.24.*X* - san.24.2 == 0 | 0.094724 | 0.024898 | 3.804 | <0.01 |
| san.18.3 - sim.24.2 == 0 | -0.10494 | 0.0226 | -4.643 | <0.01 |
| sim.18.3 - sim.24.2 == 0 | -0.036072 | 0.0226 | -1.596 | 0.9084 |
| san.24.3 - sim.24.2 == 0 | -0.116549 | 0.0226 | -5.157 | <0.01 |
| sim.24.3 - sim.24.2 == 0 | -0.02925 | 0.0226 | -1.294 | 0.9792 |
| san.18.*X* - sim.24.2 == 0 | -0.126435 | 0.024898 | -5.078 | <0.01 |
| sim.18.*X* - sim.24.2 == 0 | -0.03031 | 0.024898 | -1.217 | 0.9872 |
| san.24.*X* - sim.24.2 == 0 | -0.081335 | 0.024898 | -3.267 | 0.0499 |
| sim.24.*X* - sim.24.2 == 0 | 0.009866 | 0.024898 | 0.396 | 1 |
| sim.18.3 - san.18.3 == 0 | 0.068868 | 0.024442 | 2.818 | 0.1712 |
| **san.24.3 - san.18.3 == 0** | **-0.011609** | **0.024442** | **-0.475** | **1** |
| sim.24.3 - san.18.3 == 0 | 0.075689 | 0.024442 | 3.097 | 0.0836 |
| san.18.*X* - san.18.3 == 0 | -0.021495 | 0.026581 | -0.809 | 0.9997 |
| sim.18.*X* - san.18.3 == 0 | 0.074629 | 0.026581 | 2.808 | 0.1754 |
| san.24.*X* - san.18.3 == 0 | 0.023605 | 0.026581 | 0.888 | 0.9992 |
| sim.24.*X* - san.18.3 == 0 | 0.114806 | 0.026581 | 4.319 | <0.01 |
| san.24.3 - sim.18.3 == 0 | -0.080477 | 0.024442 | -3.293 | 0.047 |
| **sim.24.3 - sim.18.3 == 0** | **0.006821** | **0.024442** | **0.279** | **1** |
| san.18.*X* - sim.18.3 == 0 | -0.090363 | 0.026581 | -3.4 | 0.0332 |
| sim.18.*X* - sim.18.3 == 0 | 0.005761 | 0.026581 | 0.217 | 1 |
| san.24.*X* - sim.18.3 == 0 | -0.045263 | 0.026581 | -1.703 | 0.8639 |
| sim.24.*X* - sim.18.3 == 0 | 0.045938 | 0.026581 | 1.728 | 0.8519 |
| sim.24.3 - san.24.3 == 0 | 0.087299 | 0.024442 | 3.572 | 0.019 |
| san.18.*X* - san.24.3 == 0 | -0.009886 | 0.026581 | -0.372 | 1 |
| sim.18.*X* - san.24.3 == 0 | 0.086239 | 0.026581 | 3.244 | 0.0538 |
| san.24.*X* - san.24.3 == 0 | 0.035214 | 0.026581 | 1.325 | 0.9751 |
| sim.24.*X* - san.24.3 == 0 | 0.126415 | 0.026581 | 4.756 | <0.01 |
| san.18.*X* - sim.24.3 == 0 | -0.097184 | 0.026581 | -3.656 | 0.0137 |
| sim.18.*X* - sim.24.3 == 0 | -0.00106 | 0.026581 | -0.04 | 1 |
| san.24.*X* - sim.24.3 == 0 | -0.052085 | 0.026581 | -1.959 | 0.7161 |
| sim.24.*X* - sim.24.3 == 0 | 0.039117 | 0.026581 | 1.472 | 0.9466 |
| sim.18.*X* - san.18.*X* == 0 | 0.096124 | 0.02856 | 3.366 | 0.0379 |
| **san.24.*X* - san.18.*X* == 0** | **0.0451** | **0.02856** | **1.579** | **0.9144** |
| sim.24.*X* - san.18.*X* == 0 | 0.136301 | 0.02856 | 4.772 | <0.01 |
| san.24.*X* - sim.18.*X* == 0 | -0.051025 | 0.02856 | -1.787 | 0.8218 |
| **sim.24.*X* - sim.18.*X* == 0** | **0.040177** | **0.02856** | **1.407** | **0.9612** |
| sim.24.*X* - san.24.*X* == 0 | 0.091201 | 0.02856 | 3.193 | 0.063 |
